# Capivasertib restricts SARS-CoV-2 cellular entry: a potential clinical application for COVID-19

**DOI:** 10.7150/ijbs.57810

**Published:** 2021-06-11

**Authors:** Fang Sun, Chenglin Mu, Hang Fai Kwok, Jiyuan Xu, Yingliang Wu, Wanhong Liu, Jean-Marc Sabatier, Cédric Annweiler, Xugang Li, Zhijian Cao, Yingqiu Xie

**Affiliations:** 1 State Key Laboratory of Virology and Modern Virology Research Center, College of Life Sciences, Wuhan University, Wuhan, 430072, China.; 2 Sino German Joint Research Center for Agricultural Biology, and State Key Laboratory of Crop Biology, College of Life Sciences, Shandong Agricultural University, Tai'an, 271018, China.; 3 Institute of Translational Medicine, Faculty of Health Sciences, University of Macau, Avenida de Universidade, Taipa, Macau SAR; MoE Frontiers Science Center for Precision Oncology, University of Macau, Avenida de Universidade, Taipa, Macau SAR.; 4 Hubei Province Key Laboratory of Allergy and Immunology, School of Basic Medical Sciences, Wuhan University, Wuhan 430071, China.; 5 Aix-Marseille University, Institute of NeuroPhysiopathology, UMR 7051, 27, Bd Jean Moulin, 13385 Marseille cedex, France.; 6 Department of Geriatric Medicine and Memory Clinic, Research Center on Autonomy and Longevity, University Hospital, Angers, France.; 7 School of Sciences and Humanities, Biology Department, and Pilot Cluster of Multidisciplinary Comprehensive Materia Medica, Biocluster within Cluster of Life Science and Engineering at C4, Nazarbayev University, Nur-Sultan, 010000, Republic of Kazakhstan.

**Keywords:** COVID-19, SARS-CoV-2, AKT inhibitor, capivasertib, antiviral activity

## Abstract

Coronavirus disease 2019 (COVID-19) caused by severe acute respiratory syndrome coronavirus 2 (SARS-CoV-2) infection has led to more than 150 million infections and about 3.1 million deaths up to date. Currently, drugs screened are urgently aiming to block the infection of SARS-CoV-2. Here, we explored the interaction networks of kinase and COVID-19 crosstalk, and identified phosphoinositide 3-kinase (PI3K)/AKT pathway as the most important kinase signal pathway involving COVID-19. Further, we found a PI3K/AKT signal pathway inhibitor capivasertib restricted the entry of SARS-CoV-2 into cells under non-cytotoxic concentrations. Lastly, the signal axis PI3K/AKT/FYVE finger-containing phosphoinositide kinase (PIKfyve)/PtdIns(3,5)P2 was revealed to play a key role during the cellular entry of viruses including SARS-CoV-2, possibly providing potential antiviral targets. Altogether, our study suggests that the PI3K/AKT kinase inhibitor drugs may be a promising anti-SARS-CoV-2 strategy for clinical application, especially for managing cancer patients with COVID-19 in the pandemic era.

## Introduction

Pandemic coronavirus disease-2019 (COVID-19) has caused more than 150 million infections and about 3.1 million deaths up to date (https://covid19.who.int/). Although the detailed pathology and molecular mechanisms of COVID-19 are not completely clear yet, the virology investigation provided some knowledge and potential treatment avenues. The virus which causes COVID-19, named as severe acute respiratory syndrome coronavirus 2 (SARS-CoV-2), first enters into host cells by binding to the cellular receptor angiotensin-converting enzyme 2 (ACE2) via receptor-binding domain (RBD) interacting with the C-terminal domain of SARS-CoV-2 spike protein (S) 1. The binding is followed by SARS-CoV-2 S priming regulated by transmembrane protease serine 2 (TMPRSS2), which is a key factor for viral entry 1.

Upon SARS-CoV-2 virions attachment to the host cells, viral spike protein shows a conformation change which mediates the viral envelope fusion with the host cell membrane via an endosomal pathway 2. Targeting viral entry has been proposed and clinical trial has started (https://clinicaltrials.gov/ct2/show/NCT04352400) 3. However, the main consequence is the cytokine storm of the host cells upon response to infection and followed by signalling pathways transduction to the downstream targets of cell death, cell cycle and others, which may crosstalk with cancer signaling as the similar common interleukin activators 4. For example, some cytokines transduce signals through JAKs and signal transducer and activator of transcriptions (STATs) whose mislocalization or super-activation may induce cancers 5. In details, binding of interleukin to their receptors resulting in conformational changes of receptors will provide docking site for recruiting a number of signaling proteins, which may also crosstalk with kinases, such as Pim-1, Ras or AKT activation 6. In addition, other factors involved in the inflammation responses can also activate cancer-related cell proliferation signaling 7. Therefore, kinases most likely involve in the cytokine signaling of COVID-19 inflammation.

Given the similarity of signaling of cancer and COVID-19, a recent study revealed a large scale kinases mediated global phosphorylation of both viral and host cell proteins in a cell line of monkey kidney Vero E6 cells by whole proteomics analysis upon SARS-CoV-2 infection 8, 9 with cytokine storm, activating of kinases including p38 MAP kinase, CDKs, casein kinase II (CK2), and PIKFYVE kinases suggesting the potential clinical applicable kinase inhibitor drugs for the treatment of COVID-19 8. Based on different cohort of preclinical and clinical studies, it is urgently needed to analyze whole big data to get a systematic conclusion or prediction of new drugs for cancer patients infected with COVID-19. Here, we aimed to use network pharmacology tool to analyze the database of big data and explore the crosstalk pathways between whole kinases signaling and COVID-19, then to test the efficacy of the predicted drugs in a SARS-CoV-2 model system.

## Materials and Methods

### Cells

Vero cells were purchased from the China Center for Type Culture Collection (CCTCC) and were cultured in DMEM (Gibco-Invitrogen, New York, NY, USA) supplemented with 10% FBS (Gibco-Invitrogen) and 1% penicillin/streptomycin at 37 °C in an incubator filled with 5% CO_2_.

### Construction of protein-protein interaction (PPI) crosstalk-networks between kinase, drug target and COVID-19

The drug targets of COVID-19 and kinases related targets were collected by searching databases of NCBI (https://www.ncbi.nlm.nih.gov), GenCLiP3 GeneCards, using key words above and “Homo sapiens” for human species plus the input of targets from recent published pool 8 followed by the application of venny 2.1.0 tool to explore of the intersection targets, of which were exported to the STRING database to construct protein-protein networks for further analysis and visualization through Cytoscape 3.7.2 tool 10-13 (http://www.bioinformatics.com.cn). For capivasertib (AZD5363) based drug targets-COVID-19 PPI, the targets were identified based on the drug bank by dissociation constant (Kd) of 100 nM to 10 µM range of dose (https://lincs.hms.harvard.edu/db/sm/10510-101-1/), and PPIs were established after screening of crosstalk intersection targets by venny 2.1.0 tool with STRING database and through Cytoscape 3.7.2 tool as mentioned above.

### Analysis of kyoto encyclopedia of genes and genomes (KEGG) pathways and gene ontology (GO)

Target genes above were input to DAVID database and KEGG pathway analysis website for analysis of pathways by a threshold P <0.01 and online mapping was applied for visualization.

### Preparation of SARS-CoV-2 S pseudovirion and analysis of virus entry

Vesicular stomatitis virus (VSV) glycoprotein deficient VSV exogenously expressing firefly luciferase (VSV-dG-Luc) and SARS-CoV-2 pseudotyped virus whose S protein was packaged using the VSV-dG-Luc were kindly offered by Professor Huan Yan (State Key Laboratory of Virology, Wuhan University, China). VSV-dG-Luc and SARS-CoV-2 S pseudovirion were used to transduce Vero cells at a multiplicity of infection (MOI) of 1 seeded into 96-well plates. After overnight incubation with the different concentrations of a phosphoinositide 3-kinase (PI3K)/AKT inhibitor capivasertib (Beyotime Biotechnology), the firefly luciferase activity was measured by Dual Luciferase Reporter Assay Kit (DL101-01, Vazyme). Each concentration of capivasertib had 3-5 duplicates. The firefly luciferase activity in the control group without capivasertib was normalized to 1. Then, the relative luciferase activity in the group with capivasertib (0.1 μM, 1 μM and 10 μM) was calculated and drawed. The experiment was repeated at least three times.

### Target gene expression with cancer patient overall survival data-based analysis for prognosis

The database re-analysis based on the transcripts per kilobase million (TPM) was performed for patient overall survival according to instructions as described in the website 14. The poor outcomes of prognosis were identified and non-related outcomes were not further analyzed.

## Results

### Network pharmacology analysis identified AKT signaling as potential top ranked kinase crosstalk with COVID-19 pathways

**Figure 1:**
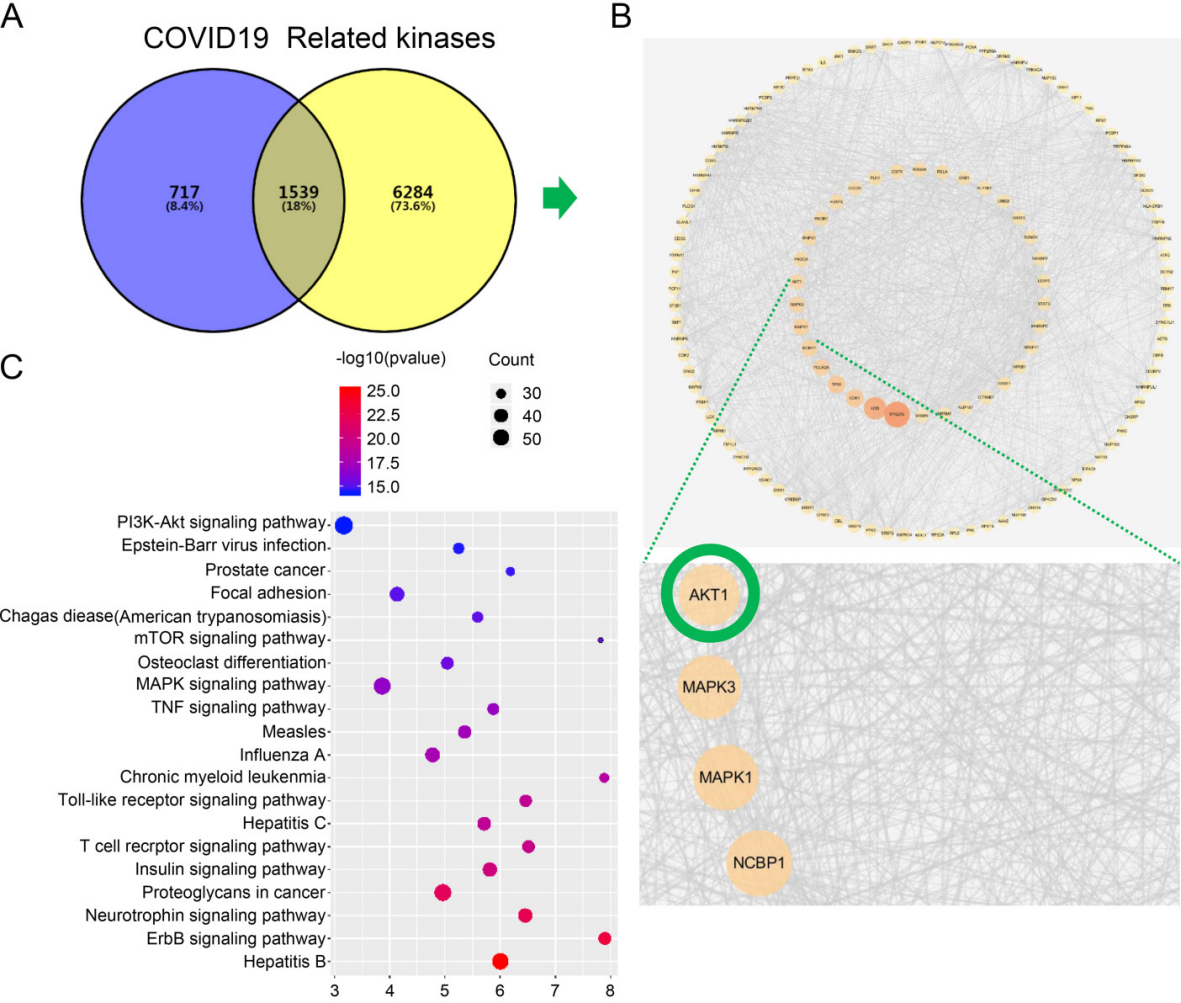
** Network pharmacology identified the kinase signaling crosstalk with the COVID-19. A.** Analysis of the intersection target genes by venny 2.1.0 tool. The Venn diagram represents the number of intersection of the COVID-19 target and the kinase-related genes. In order to distinguish the two sets of data, different colors are used as the standard. The size of the circle does not represent the proportion, but the proportion of different parts. The purpose is to show the proportion of the data set occupied by the intersection genes. To show the intersection result more clearly, we have modified the color of the text on the graph. **B.** Establishment protein-protein interaction (PPI) crosstalk-networks between kinase and COVID-19 were constructed as described in the methods. The network of the interaction score was set to 0.9 for screening, and 1244 nodes and 12426 edges were obtained. The average degree value is 20, and two times the average degree value is selected as the screening condition. The points with a degree value ≥ 40 are used to visualize the protein interaction network with cytoscape. The size of the node, and the intensity of color, shows the degree of value. Each edge represents the interaction between proteins. The line number indicates the degree of association. **C.** Kyoto encyclopedia of genes and genomes (KEGG) pathways indicating AKT pathway is the top ranked.

**Figure 2:**
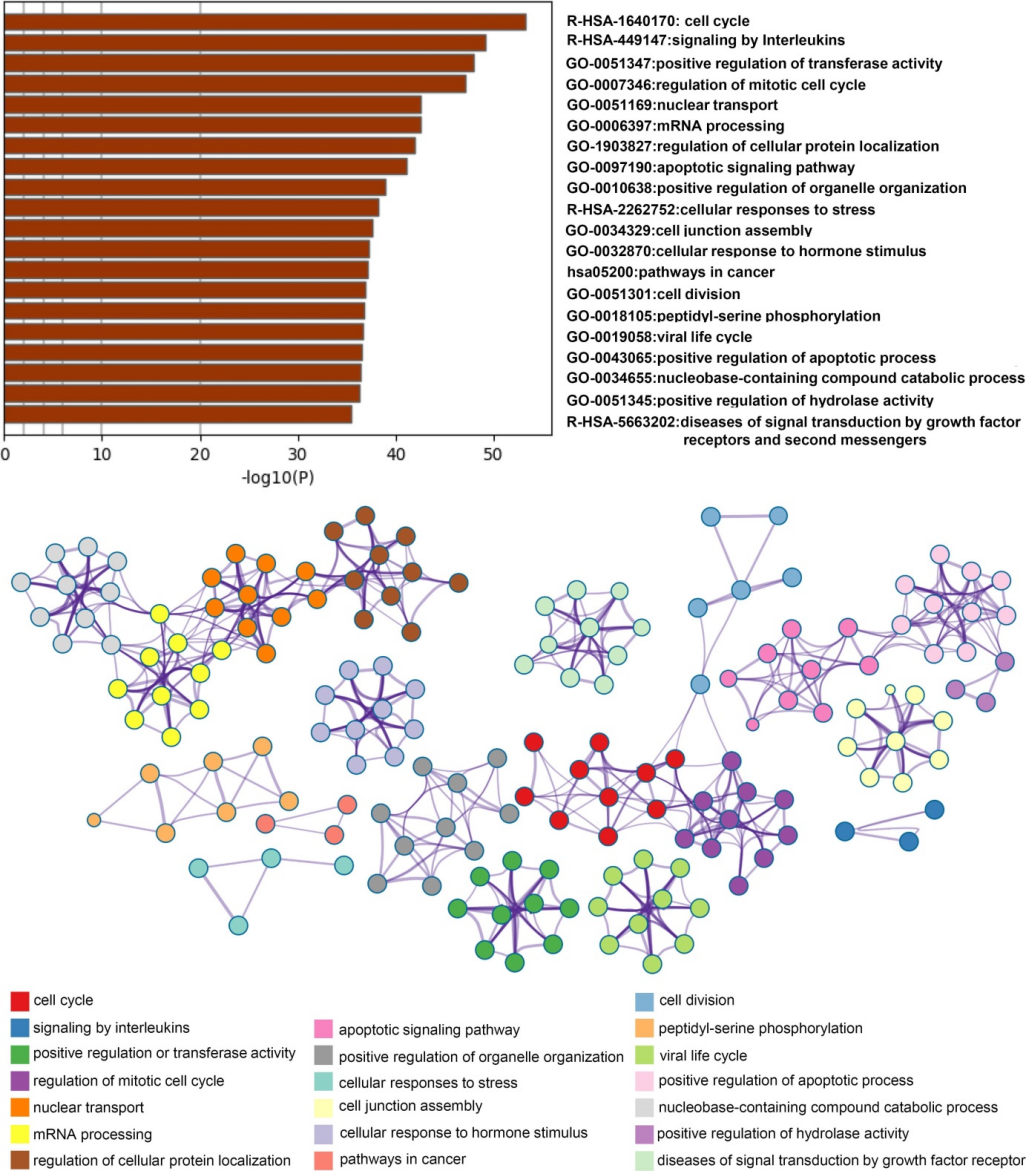
** Analysis of network related biological functions.** Identified statistically enriched items were clustered as a tree by Kappa-statistical similarities and 0.3 kappa score of the threshold was used for clusters followed by converting into network small maps with colors indicating groups. The network was shown with Cytoscape (v3.1.2) and items description indicated.

Firstly, we searched a variety of databases for “COVID-19” and “kinase” targets from NCBI (https://www.ncbi.nlm.nih.gov), GenCLiP3 (http://ci.smu.edu.cn/genclip3/GeneAssociation.php), GeneCards (https://www.genecards.org/), databases, and Venny 2.1.0 (https://bioinfogp.cnb.csic.es/tools/venny/) tool for mapping the common targets. We obtained the predicted encoding intersection targets suggesting the interaction proteins involved in kinase mediated COVID-19 infection, progression, and host cell reaction (Figure 1A). Using the screened 1539 co-targets (18%), we then established the protein-protein interaction map and analyzed the most essential pathways by nodes, GO and KEGG of top ranking of gene counts (Figure 1C and Figure 2). The top ranked kinases are consistent with the recent publication, such as CDK, p38/MAPK kinases serving as the positive control (Figure 1B) 8. The new finding is that PI3K/AKT kinase is in the top ranked kinase list (Figure 1B). Moreover, the GO and KEGG data showed that top ranked pathways are involving the cytokine, cell cycle, cancer, mRNA processing, and viral life cycle (Figure 1C and Figure 2). Thus, our network pharmacology analysis suggests that AKT may be the potential kinases associated with the COVID-19 disease.

### AKT inhibition restricts SARS-CoV-2 entry

**Figure 3:**
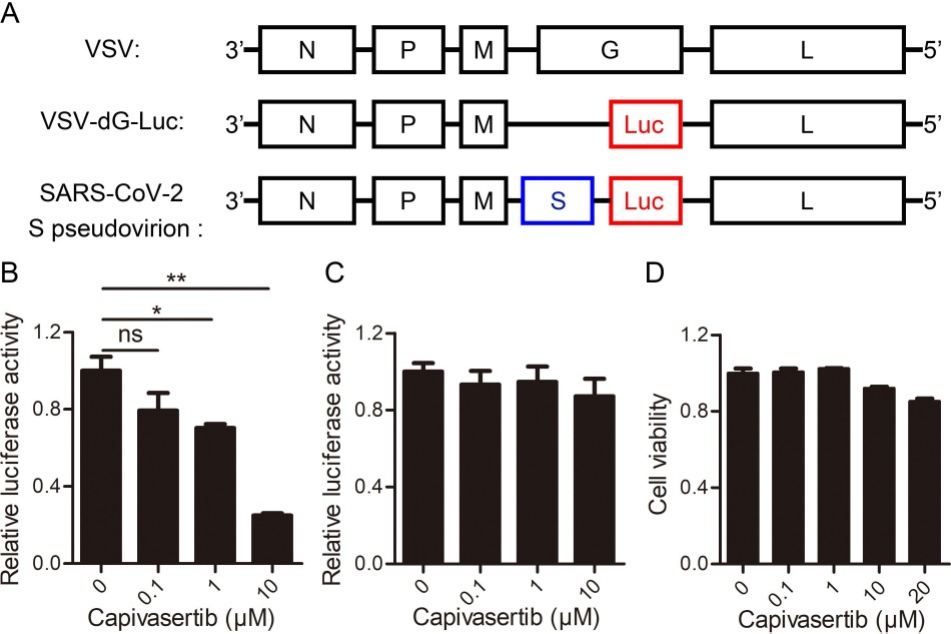
** Inhibitory effects of AKT signal inhibitor on the entry of SARS-CoV-2 to cells with a dose-dependent manner under non-cytotoxic concentrations. A.** The negative-strand genome model of VSV, VSV-dG-Luc, and SARS-CoV-2 S pseudovirion. Black boxes represent five genes of VSV, which encode nuclear protein (N), phosphate protein (P), matrix protein (M), glycoprotein protein (G), and RNA polymerase (L), respectively. Red box represents firefly luciferase gene, and blue box represents glycoprotein gene of SARS-CoV-2. **B. and C.** Concentration-dependent inhibition of capivasertib on SARS-CoV-2 entry into Vero cells. Vero cells infected by SARS-CoV-2 S pseudovirion (B) and VSV-dG-Luc (C) at an MOI of 1 were incubated with different concentrations of capivasertib (0, 0.1 μM, 1 μM, and 10 μM). After 20 h, cells were collected and intracellular luciferase activity was analyzed by luciferase reporter assay. **D.** Cytotoxicity of capivasertib to Vero cells by the MTT assay. ns, no significance. *, p < 0.05. **, p < 0.01.

**Figure 4:**
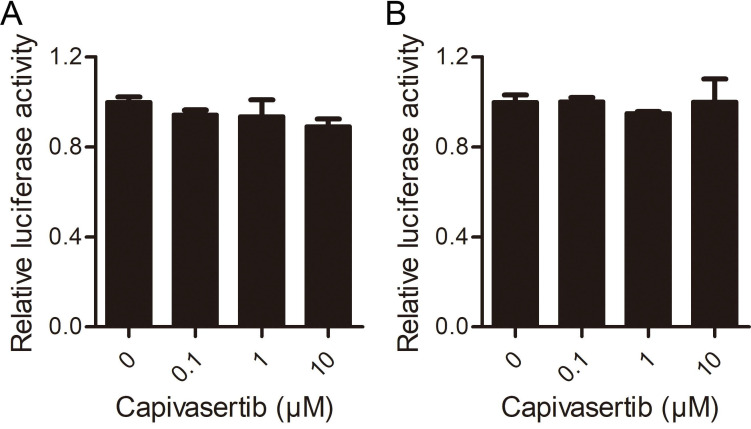
** Effects of capivasertib on VSV-dG-Luc in Vero cells with SARS-CoV-2 spike overexpression or not. A. and B.** Control vector pCAGGS (A) and the recombinant vector pCAGGS-HA-spike (B) were transfected into Vero cells for 24 h, respectively. Then, cells infected by VSV-dG-Luc at an MOI of 1 were incubated with different concentrations of capivasertib (0, 0.1 μM, 1 μM, and 10 μM), respectively. After 20 h, cells were collected and intracellular luciferase activity was analyzed by luciferase reporter assay.

Next, we aimed to explore the influence of AKT kinases signaling on COVID-19 by testing the efficacy of the predicted drug in a SARS-CoV-2 model system. Capivasertib is a potent pan-AKT kinase inhibitor drug that inhibits AKT1, AKT2 and AKT3. Capivasertib had significant antitumor activities, and was being used as an oral small-molecule AKT inhibitor for drug-resistant breast cancer in clinical trials 15, 16. Here, we explored the influence of capivasertib on SARS-CoV-2 S protein pseudotyped virus and VSV-dG in Vero cells by the luciferase report assay. The negative-strand genome models of VSV, VSV-dG-Luc, and SARS-CoV-2 S pseudovirion were shown in Figure 3A. The quantification analysis showed that capivasertib had a concentration-dependently inhibitory activity against intracellular luciferase activity of SARS-CoV-2 S pseudovirion and the 50% inhibitory concentration was 2.33 ± 1.40 μM (Figure 3B), while capivasertib (0.1, 1 and 10 μM) had no inhibitory activity on the infection of VSV-dG-Luc (Figure 3C). In addition, the treatment of capivasertib (0.1, 1 and 10 μM) did almost not affect the growth and survival of Vero cells, but 20 μM capivasertib can inhibit 20% Vero cells (Figure 3D). The 50% cytotoxic concentration of capivasertib to Vero cells was 82.1 ± 1.52 μM. The selection index of capivasertib was about 35, which indicated that capivasertib was relatively safe *in vitro*. In fact, luciferase activity could reflect viral infection rate changes from two steps of viral entry and viral genome replication/expression. In order to exclude the role of capivasertib during the step of viral genome replication/expression, we tested the effect of capivasertib on VSV-dG-Luc in Vero cells where SARS-CoV-2 spike was overexpressed or not. The data indicated that the treatment of capivasertib had no effect on the luciferase activity of VSV-dG-Luc infecting Vero cells with SARS-CoV-2 spike overexpression or not, suggesting that capivasertib was not related to the genome replication and expression of VSV-dG-Luc (Figure 4). Clearly, these results suggest that capivasertib, an AKT-targeted anti-cancer drug, possibly restricts the entry of SARS-CoV-2 to cells under non-cytotoxic concentrations and has a great potential for clinical trial of anti-SARS-CoV-2.

### AKT profiling in cancer overall survival

**Figure 5:**
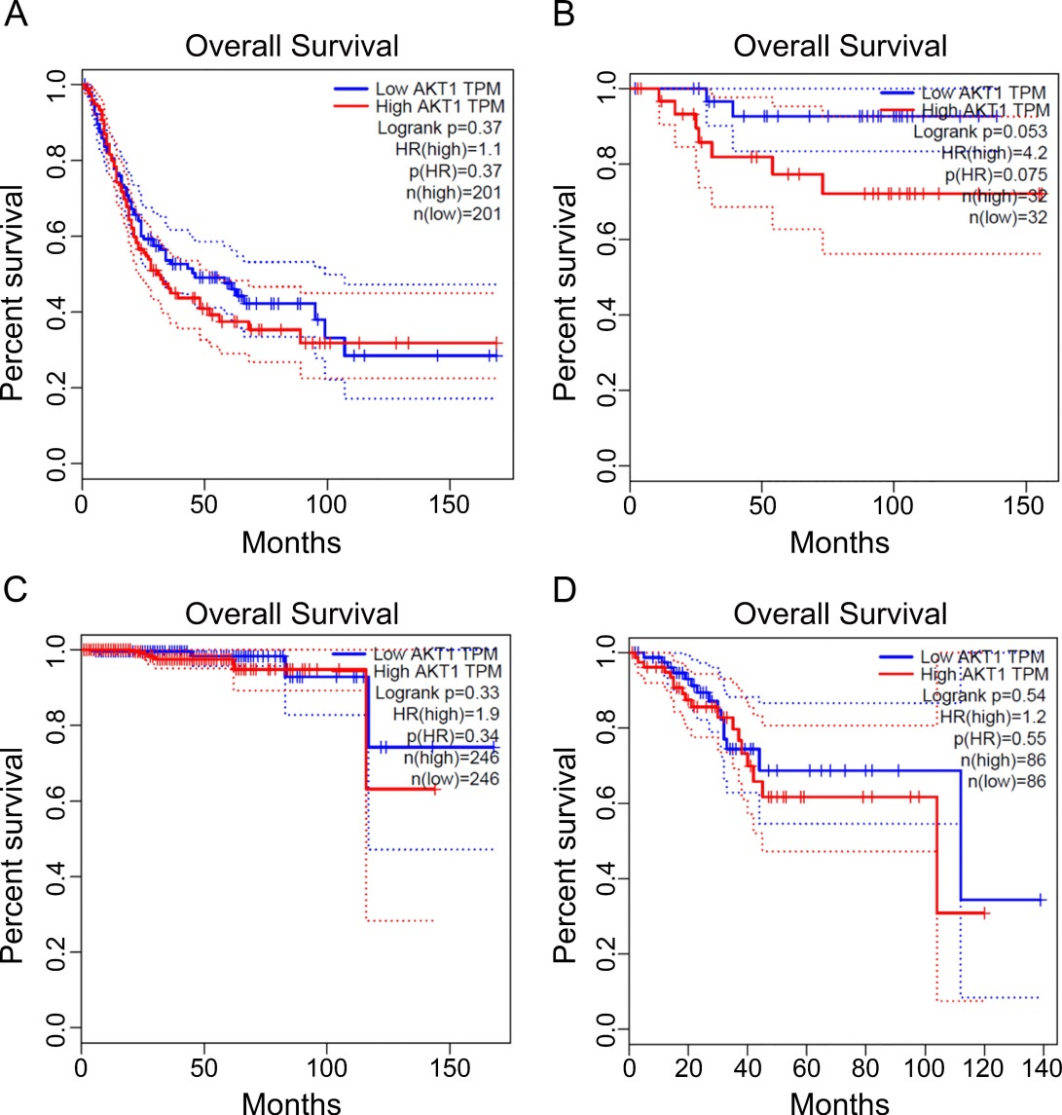
** The prognosis of the identified crosstalk targets in cancers.** The transcripts per kilobase million (TPM) of specific genes listed were analyzed for patient survival in different cancers. The cancer types are from A to D: urothelial bladder carcinoma, kidney chromophobe carcinoma, prostate adenocarcinoma, and uterine corpus endometrial carcinoma. The data were obtained as described in methods using GEPIA online analysis tool 20 for *AKT1* prognostic analysis.

Finally, we searched database of gene expression profiling of the targets and listed the poor outcome of overall survival which correlated to the gene overexpression encoding for AKT kinases. The significant cancer types are urothelial bladder carcinoma, kidney chromophobe carcinoma, prostate adenocarcinoma, uterine corpus endometrial carcinoma for AKT (Figure 5). Thus, our data suggest the identified kinase network related COVID-19 targets has poor prognosis in some types of cancer. The AKT inhibitor drugs, such as capivasertib, could be recommended for concurrent targeting both COVID-19 and the type of cancer in the pandemic era for unique treatment.

## Discussion

Here, we reported a whole kinase-protein-protein interaction network, which identified a top ranked AKT kinase-mediated pathway and the related clinical anti-cancer drugs for possibly treating COVID-19 patients by targeting AKT. The clinical applicable drug capivasertib has a great potential for clinical trial of anti-SARS-CoV-2.

**Figure 6:**
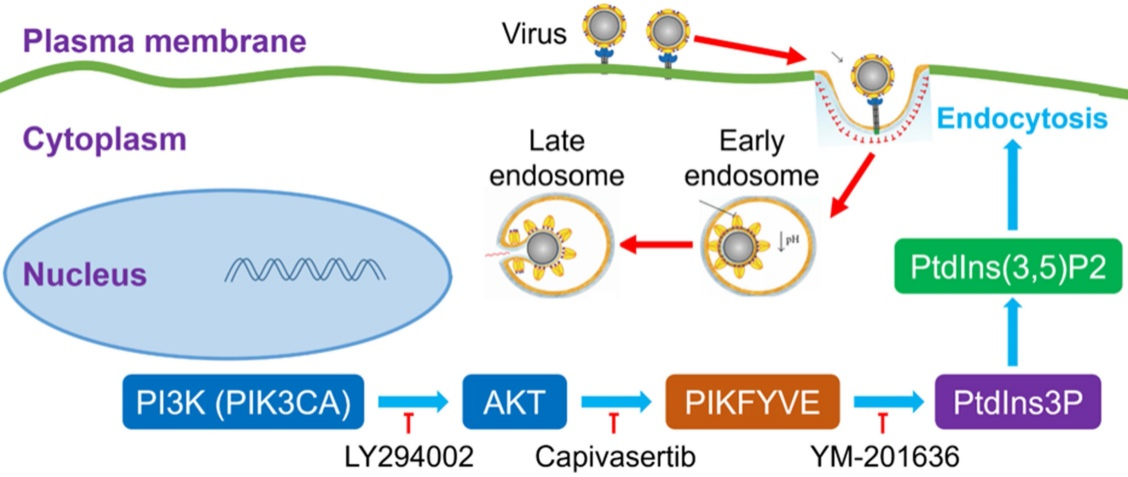
**An intracellular pathway model for pharmacological blockage of PI3K(PIK3CA)/AKT signal axis restricting the entry of viruses including SARS-CoV-2.** 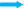 activation or promotion; 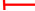 inactivation or inhibition; PI3K, phosphatidylinositol 3-kinase; PIK3CA, phosphatidylinositol 3-kinase catalytic subunit α; AKT, serine/threonine kinase (PKB, protein kinase B); PIKFYVE, FYVE finger-containing phosphoinositide kinase; PtdIns3P, phosphatidylinositol-3-phosphate; PtdIns(3,5)P2, phosphatidylinositol-3,5-biphosphate.

It was very interesting to investigate the detailed relationship between PI3K/AKT signal pathway and viral infection. The previous many studies reported that the activation of the PI3K/AKT signaling pathway facilitated viral replication 17, 18, which suggested that our finding about the inhibitor of PI3K/AKT restricting SARS-CoV-2 entry to cells was not just one case. The PI3K/AKT signal pathway possibly played a common role during many viral infections. Very importantly, PI3K(PIK3CA)/AKT cascade reaction can activate a FYVE finger-containing phosphoinositide kinase (PIKfyve), whose principal enzymatic activity is to phosphorylate PtdIns3P to PtdIns(3,5)P2 17, 18. PIKfyve participates in several aspects of endosome dynamics via PtdIns(3,5)P2 production. In fact, the endocytic pathway is one of the most important ways for viruses to enter host cells. It was easily believed that the interference of PI3K/AKT/PIKfyve/PtdIns(3,5)P2 signal axis could regulate viral replication. Indeed, a PI3K inhibitor LY294002 was reported to effectively decrease the replication and DNA synthesis of Marek's disease virus (MDV) 17, and a highly selective PIKfyve inhibitor YM-201636 was also found to inhibit retrovirus replication 18. Therefore, the signal pathway of PI3K/AKT/PIKfyve/PtdIns(3,5)P2 would provide potential druggable targets for many viruses with the endocytic pathway for entering cells (Figure 6).

PI3K/AKT signaling plays a key role in cell proliferation, survival, growth, migration, invasion, and can inhibit apoptosis and promote angiogenesis. Abnormal PI3K/AKT signaling pathway can cause diseases, such as cancer. Cancer has always been one of the major health problems. With the continuous progress of life science, targeted therapy has become the latest popular method for malignant tumor. Among them, inhibitors of the PI3K/AKT pathway play an essential role in cancer treatment. PI3K and AKT are potential tumor drug targets, and their anti-tumor therapies show attractive prospects. Capivasertib is a potent pan-AKT kinase inhibitor drug that inhibits AKT1, AKT2 and AKT3. It had significant antitumor activity and was an attractive lead of antitumor drugs. Capivasertib is being used as an oral small-molecule AKT inhibitor for drug-resistant breast cancer in clinical trials 19, 20, 21. Thus, targeting the PI3K/AKT by concurrent anti-cancer and anti-viral drugs would provide potential of the best management of cancer patients in the COVID-19 era.

## Conclusion

Our study showed that a kinase inhibitor capivasertib, also an AKT-targeted anti-cancer drug, could prevent the entry of SARS-CoV-2 to cells. There are so many antitumoral drugs targeting PI3K/AKT signal pathway in clinical trials, which would possibly be valuable drug resources to combat the current COVID-19 pandemic and be worth managing the critical urological cancer patients with COVID-19 in the pandemic era.

## Abbreviations

COVID-19: coronavirus disease-2019
SARS-CoV-2: severe acute respiratory syndrome coronavirus 2
PPI: protein-protein interaction network
ACE2: angiotensin-converting enzyme 2
TMPRSS2: transmembrane protease serine 2
S: spike protein
PI3K: phosphoinositide 3-kinase
RBD: receptor-binding domain
STATs: signal transducer and activator of transcriptions
CK2: casein kinase II
Kd: dissociation constant
KEGG: kyoto encyclopedia of genes and genomes
GO: gene ontology
PIKfyve: FYVE finger-containing phosphoinositide kinase
MDV: Marek's disease virus
TPM: transcripts per kilobase million
VSV: vesicular stomatitis virus
VSV-dG-Luc: glycoprotein deficient VSV exogenously expressing firefly luciferase
MOI: multiplicity of infection
